# Toxicological assessment of multi-walled carbon nanotubes *in vitro*: potential mitochondria effects on male reproductive cells

**DOI:** 10.18632/oncotarget.9689

**Published:** 2016-05-29

**Authors:** Cheng Xu, Qian Liu, Hui Liu, Chunlan Zhang, Wentao Shao, Aihua Gu

**Affiliations:** ^1^ State Key Laboratory of Reproductive Medicine, Institute of Toxicology, Nanjing Medical University, Nanjing, China; ^2^ Key Laboratory of Modern Toxicology of Ministry of Education, School of Public Health, Nanjing Medical University, Nanjing, China

**Keywords:** multiwalled carbon nanotubes, in vitro, male reproduction, spermatocyte, mitochondria, Pathology Section

## Abstract

Multi-walled carbon nanotubes (MWCNTs) have been widely used in many fields and were reported to cause reversible testis damage in mice at high-dose. However the reproductive effects of low dose MWCNTs remained elusive. Herein, we used the mice spermatocyte cell line (GC-2spd) to assess the reproductive effects of MWCNTs. Size distribution, zeta potential, and intensity of MWCNTs were characterized. A maximal concentration of 0.5 μg/mL MWCNTs was found to be nonlethal to GC-2spd. At this dose, cell cycles and the ROS levels were in normal status. We also found MWCNTs accumulated in mitochondria, which caused potential mitochondrial DNA damage in spermatocyte. Furthermore, the expression level of mitochondria-related genes, the oxygen consumption rate, and cellular ATP content were declined compared to controls, even at the nonlethal dose. Our results suggested for the first time that, in germ cells, mitochondrion was a cellular organelle that accumulated MWCNTs.

## INTRODUCTION

Carbon nanotubes (CNTs) are allotropes of carbon with large length-to-diameter ratio. In general, CNTs are categorized as single-walled CNTs (SWCNTs) and multi-walled CNTs (MWCNTs). MWCNTs have recently been applied in wide fields such as composite material development, medical contrast imaging agents, and drug delivery [1–3]. However, the effects of MWCNTs on human health are still unclear due to limit epidemiologic information. Therefore, *in vitro* and *in vivo* data are useful. Our study focused on the effect of low dose of MWCNTs exposure on male reproductive system. To our knowledge, no similar study has ever been reported before. Although MWCNTs were considered safe for use [4], the potential toxicity has been reported in several recent studies [5–7], mainly on the genotoxicity and carcinogenicity [8–10]. Meanwhile, researchers paid more attentions on the biological effects of the MWCNTs, and serial low concentrations of MWCNTs including 12.5 μg/mL [7], 5 μg/mL [11], 5 mg/mL [12], 2.5 μg/mL [13] for different kinds of cell lines were adopted. A recent study evaluating the safety of MWCNTs for health risk with *in vivo* dose (i.t. administration, 1 mg/kg b.w., in rats) and *in vitro* dose (exceedingly low concentration of 1 μg/mL, in human A549 pneumocytes) still showed potential pulmonary toxic effects [14]. Compared with all those doses used, 1 μg/mL MWCNT was the lowest concentration in all the studies ever reported.

However, little information was available on the male reproductive hazards of MWCNTs exposure. One previous study found that MWCNTs partially damaged seminiferous tubules with increasing ROS levels and cytotoxicity [15]. Although the repeated carbon nanotube administrations in male BALB/c mice seemed not impair fertility, while the intravenously injection of 5 mg/kg nanotubes still cause oxidative stress and reversible testis damage [15]. Actually, the reproductive effects of low-dose MWCNTs are more important than that of high-dose MWCNTs, which remains unclear yet. In this study, the GC-2spd cell line, which derived from immortalized mouse spermatocyte, was used as an available in vitro model of germ cells, to address two important and challenging issues: (i) The potential reproductive effects of nonlethal-dose MWCNTs exposure, and (ii) an effectively in vitro assay to evaluate the potential reproductive effects, and the *in vitro* sensitive markers as well.

## RESULTS

### Characterization of MWCNTs

**Figure 1:**
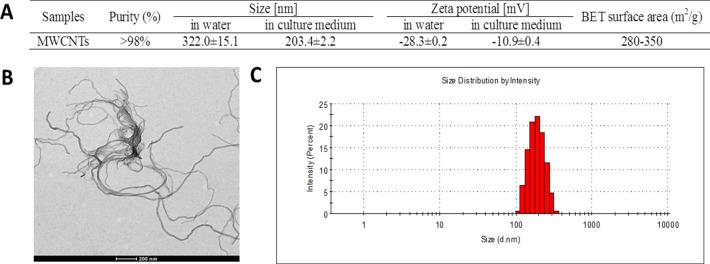
Characterization of MWCNTs **A**. General characteristic and particle-size and zeta potential of MWCNTs in water and in cell culture medium. **B**. TEM image of MWCNTs, bar = 200 nm. **C**. Particle-size distribution of MWCNTs prepared in cell culture medium.

The characteristics of MWCNTs in double distilled water and cell culture medium were showed in Figure 1A. The size of MWCNTs appearing like approximate tubulose was demonstrated by TEM (Figure 1B), which was consist with the specification sheet. Because serum proteins have better dispersion property for MWCNTs [16], the particle size distribution (Figure 1C) showed that MWCNTs had a lesser particle-size distribution of approximate 180 nm in culture medium than about 300 nm in double distilled water. The zeta potential of MWCNTs was −10.9 ± 0.4 mV in cell culture medium, and −28.3 ± 0.2 mV in double distilled water, respectively.

### The effects of MWCNTs on GC-2spd cell viability

**Figure 2:**
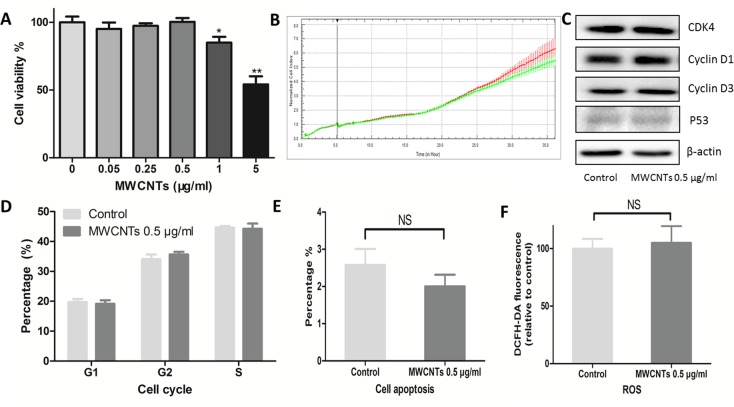
Cytotoxic responses of GC-2spd cells to MWCNTs **A**. Viability of GC-2spd cells treated with different concentrations of MWCNTs for 24 h was measured using alamarBlue method. **B**. Viability of GC-2spd cells was examined by xCELLigence approach. The red and green line represent viability of control and 0.5 μg/mL MWCNTs treated, respectively. **C**. GC-2spd cells were incubated with MWCNTs (0.5 μg/mL) for 24 h, the expression levels of CDK4, Cyclin D1, Cyclin D3, and P53 were determined by western blotting. **D**. Cell cycle profile were determined via PI staining using flow cytometer. **E**. After incubating with MWCNTs (0.5 μg/mL) for 24 h, GC-2spd cells were were labelled with annexin V-FITC and DAPI using flow cytometer. **F**. The ROS levels were assessed by flow cytometer after MWCNTs exposure to GC-2spd cells and stained with DCFH-DA. These values were all expressed as means ± S.E. from three separate experiments. * represented as *P*<0.05, ** represented as *P*<0.01, NS represents no significance.

GC-2spd cells were treated with various concentrations of MWCNTs (0, 0.05, 0.25, 0.5, 1, and 5 μg/mL) for 24 h before cell viability detection. Considering that nanoparticles may influence the accuracy of cell viability analysis [17], we used both alamarBlue method and xCELLigence approaches to obtain and confirm the cell viability results. Compared with control, no remarkable cytotoxicity was observed in GC-2spd cells treated with 0.5 μg/mL of MWCNTs for 24 h (Figure 2A, 2B, Figure S1), suggesting that concentration was a relatively safe dose, which was double confirmed by the following data: compared with control, the key markers of cell cycle and cell apoptosis were determined by western blotting (Figure 2C), and neither impair cell cycle (Figure 2D) nor cell apoptosis (Figure 2E) were changed in the cells treated with 0.5 μg/mL of MWCNTs. Furthermore, we did not find increased ROS level in GC-2spd cells treated with the dose of 0.5 μg/mL (Figure 2F). Therefore, 0.5 μg/mL was considered as a non-lethal dose of MWCNTs to GC-2spd cell viability.

### MWCNTs accumulated in mitochondria of GC-2spd cell

**Figure 3:**
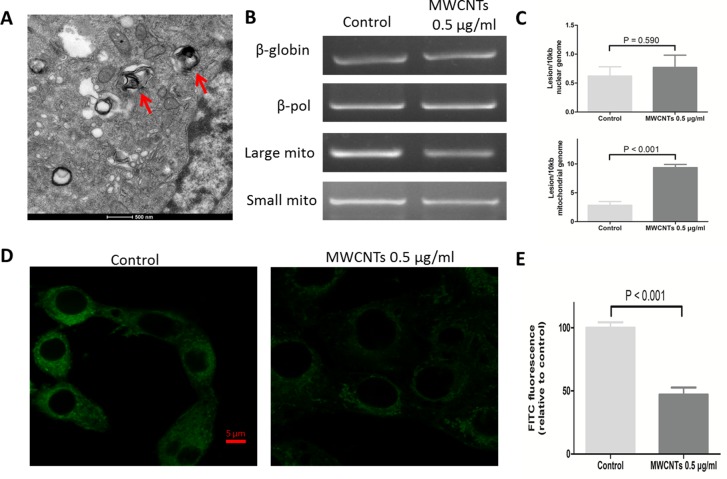
The effect of MWCNTs on the mitochondrial organelle in GC-2spd cells **A**. TEM image showed a section of GC-2spd cells treated with MWCNTs. MWCNTs were accumulated in mitochondria of GC-2spd. **B**. The electrophoretic results of nuclear genomic DNA fragments (i.e. nDNA) and mitochondrial genomic DNA fragments (i.e. mtDNA) in GC-2spd cells. **C**. Quantitative levels of damage to nDNA and mtDNA of the GC-2spd cells between MWCNTs treatment and controls were shown on each right side. **D**. The labeling of mitochondria in GC-2spd cells. Confocal microscopy images showing the mitochondria amount and location after MWCNTs exposure. **E**. Quantitative levels of mitochondria fluorescence intensity. Each data was represented as the means ± S.E. from three separate experiments.

To explore the possible subcellular localization and potential adverse effect caused by MWCNTs, the uptake of MWCNTs was imaged by TEM, which showed that, MWCNTs accumulated in the mitochondria of GC-2spd cell (Figure 3A). We then detected the potential effect on mitochondria by long PCR approach [18] and found that the nonlethal-dose MWCNTs did not damage nuclear DNA (nDNA) genome, but increased mitochondrial DNA (mtDNA) lesions compared to control group (Figure 3B, 3C). Furthermore, we measured fluorescence intensity of mitochondria itself by stain with Mito-tracker Green dye, some mitochondria lost the green fluorescence, which was different from the control group (Figure 3D, 3E).

### The effects of MWCNTs on mitochondrial function of GC-2spd cell

**Figure 4:**
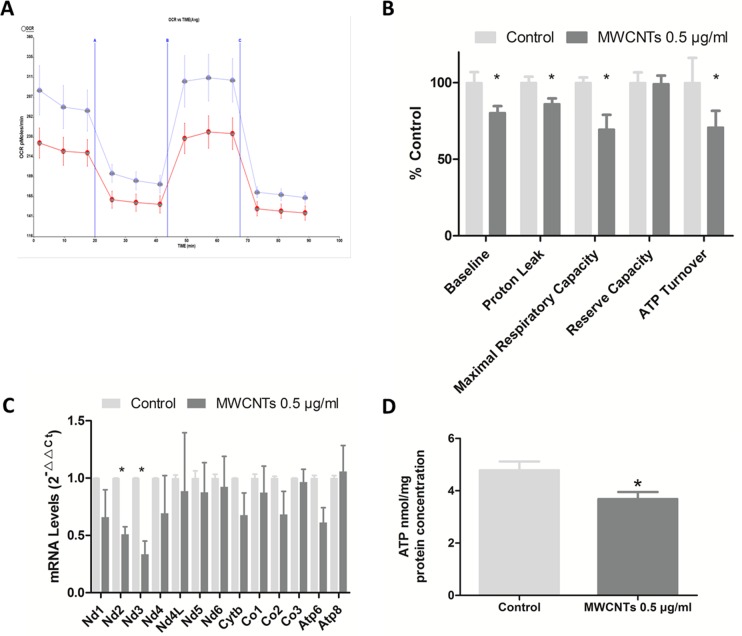
The mitochondrial functions after MWCNTs exposure to GC-2spd cells **A.** Effects of MWCNTs on cellular OCR (oxygen consumption rate) were measured with the XF96 Extracellular Flux Analyzer. GC-2spd cells were respectively incubated with control or 0.5 μg/mL for 24h. Three inhibitors, oligomycin (1 μM), FCCP (0.75 μM) and antimycin A/rotenone (0.5 μM each), were added at the different stage. The four periodes represent cellular endogenous rate, ATP-synthase-inhibited rate maximal uncoupled rate, and rotenone- and antimycin-A-inhibited rate. The results were normalized with cell protein levels. The blue and red lines respectively represent control and treated with 0.5μg/mL MWCNTs. **B.** Quantitative histogram of OCR results. **C.** RT-qPCR analysis of the expression of genes between two groups in GC-2spd cells. The *18S rRNA* was used as internal control. **D.** The ATP levels of GC-2spd cells detected by fluorescence reaction approach, adjusted by cell protein levels. *, *p* value < 0.05. Values were expressed as means ± S.E. from three separate experiments.

Oxygen consumption is at the upstream of intracellular ATP producing process, and oxygen consumption rate is one index of the mitochondrial function. We found that oxygen consumption rate of GC-2 spd cells decreased after MWCNTs treatment, with basal respiration rate significantly decreased 19.7% in 0.5 μg/mL MWCNTs group than that in control group, after adjusted by cells protein concentration (Figure 4A, 4B). Furthermore, the proton leak, maximal uncoupler-evoked oxygen consumption rate (reflecting maximal respiratory capacity), and ATP turnover respectively decreased 23.9%, 30.5%, and 29.2% in 0.5 μg/mL MWCNTs group respectively, when compared with control groups. But the cellular reserve capacity was not altered.

A total of 13 genes encode proteins involving in mitochondrial respiratory chain on mammalia mitochondrial genome [19], and the expression level of these genes in GC-2spd cells treated with 0 and 0.5 μg/mL MWCNTs were also detected in our study. We found that the expression levels of MT-ND2 (mitochondria-NADH dehydrogenase subunit 2) and MT-ND 3 (mitochondria-NADH dehydrogenase subunit 3) declined in 0.5 ug/mL MWCNTs group compared to control (Figure 4C).

ATP, the molecular unit of currency of intracellular energy transfer, was mostly produced in mitochondria by aerobic respiration in cells. ATP content was detected in GC-2spd cells with non-lethal dose MWCNTs treatment, which was lower than control (P<0.05) after adjusted cellular protein concentration (Figure 4D).

## DISCUSSION

**Figure 5:**
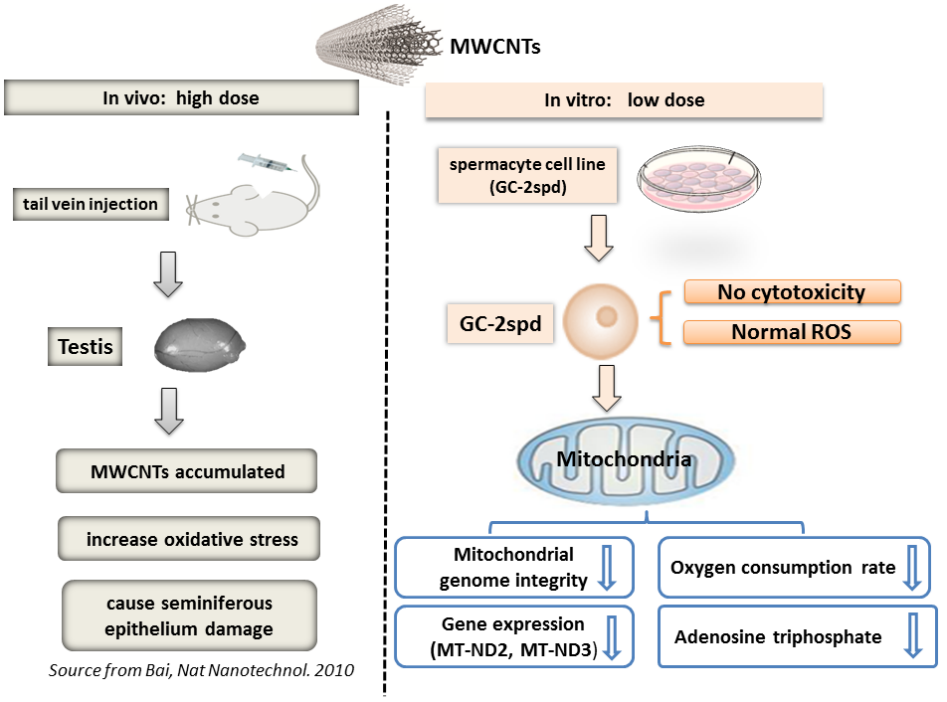
Schematic diagram showed the effects of MWCNTs on male reproduction (***in vivo*** with high dose, Source from Bai, *Nat Nanotechnol*. 2010) and (*in vitro* with low dose, The present study).

In our study, we demonstrated firstly that, 0.5 μg/mL MWCNTs, was the ‘maximum’ but ‘non-lethal’ dose without disturbing the health status of spermatocyte cell line. It had been 5 years since the MWCNTs was reported to penetrate blood-testis barrier and cause reproductive toxicity in mice at a high concentration (5mg/kg, injections via the tail vein, described in a diagram, Figure 5) [15]. But little information about the effects of non-lethal dose MWCNTs on male reproduction was available so far due to lack of an effective *in vitro* model. Herein, GC-2spd cells were used to mimic spermatocyte to investigate the biological effects and genotoxicity of the non-lethal dose MWCNTs.

Previous studies reported that, respiratory epithelial cells exposed to MWCNTs at the concentration up to 24 μg/mL, the cell viability was not changed [20]. Similarly in macrophage like (THP-1), small airway epithelial (SAE), and intestinal (Caco-2/HT29-MTX) cells, the ‘maximum’ but ‘nonlethal’ dose of MWCNTs was all up to 10 μg/mL [21]. Herein, in GC-2spd cells, even at concentration of 1 μg/mL, we still found the cell viability decreased 15% compared to control groups, suggesting the sensitivity of germ cells to damage by MWCNTs exposure.

Previous study also reported that, DNA damage occurred in mouse embryonic stem cells [22] treated with 100 μg/mL MWCNTs for 24 h. Our data showed that, under a non-lethal dose of 0.5 μg/mL MWCNTs, mitochondrial DNA damage could already be observed before occurring of nDNA impairment in cells with health status, which indicated that mtDNA was more sensitive to MWCNTs compared with nDNA. The reasons might lie in three points: (1) lack of protective histones and proximity ROS originating from mitochondria [23]; (2) intact DNA damage repair in mitochondria and (3) accumulating of MWCNTs in mitochondria rather in nucleus. Therefore, the data indicated that mtDNA damage could be used as a sensitive biomarker superior to cell viability and nDNA status in the safety assessment of low doses MWCNTs exposure.

It is known that excessive increasing ROS levels may result in mitochondrial dysfunction [24] and even mtDNA lesion [25]. In this study, we measured ROS levels in GC-2spd cells after MWCNTs exposure for 24 h, to explore whether ROS contributed to the mtDNA damage. We found the non-lethal dose did not increase ROS concentrations, in contrast to previous findings that MWCNTs could increase ROS levels in human small airway epithelial cells (exposure to 1.2 μg/mL) [26]. It seemed that the ROS levels in cells treat with 0.5 μg/mL MWCNTs did not account for the mtDNA damage. The mtDNA lesion might be caused by “physical trauma” of MWCNTs, which was consisted with the phenomenon of the MWCNTs accumulation in mitochondria [27]. It was also observed in colon cells that, transport activity reduced without increasing ROS levels in MWCNTs exposure group [28], which according with our findings indirectly. Thus we speculated that the disturbed the mitochondrial structure may be the reason of mitochondiral dysfunction.

In conclusion, we applied spermatocyte cell as *in vitro* model to investigate the biological effects of the non-lethal dose of 0.5 μg/mL MWCNTs (summarized in a diagram, Figure 5). In this study, we found that, compared with the previous *in vitro* studies, germ cell (GC-2spd) was more sensitive to MWCNTs than other cell lines, as the maximum non-effective dose in cell vitality was much lower than that of other cells. We also found that, in germ cell (GC-2spd), mitochondria were more sensitive to MWCNTs than other organelles. Before cellular ROS and other cellular indicators changed, the related gene expressions and functions of mitochondria had altered in response to the extremely low dose MWCNTs exposure stress. Our study was also expected to shed light on the effect of low dose MWCNTs on male reproduction and provide several worthy information for MWCNTs safe application.

## MATERIALS AND METHODS

### The characteristics and dispersion of MWCNTs

MWCNTs (Sigma-Aldrich, 773840, 4.5 nm ± 0.5 nm × 3~6 μm, Saint Louis, MO, USA) were characterized both in double distilled water and in complete cell culture medium contained with 10% fetal bovine serum (FBS, Sigma-Aldrich, Saint Louis, MO, USA). A JEOL JEM 2100 transmission electron microscope was used to image the MWCNTs. The MWCNTs size distributions and the zeta potential were analyzed in a Zetasizer Nano series model ZS (Brookhaven Instrument Corp.).

### Cell culture and MWCNTs treatment

The immortalized cell lines GC-2spd(ts) produced by transformation with the SV40 large T antigen GC-2spd was purchased from ATCC (CRL-2196, Manassas, VA, USA) and cultured at 37°C, 5% CO_2_ with dulbecco's modified eagle medium, contained with 10% FBS, 100 U/mL penicillin(Sigma-Aldrich, Saint Louis, MO, USA), and 100 mg/mL streptomycin(Sigma-Aldrich, Saint Louis, MO, USA). When the cell confluency reached 50%, the freshly ultrasonic MWCNTs were added and incubated for 24 h.

### Cell viability assay

Cells were plated on 96-multi-well plates and administrated with different concentrations (0, 0.05, 0.25, 0.5, 1, 5 μg/mL) of MWCNTs for 24 h. Then, a total of 20 μl alamarBlue (Thermo Fisher Scientific, Carlsbad, CA, USA) per well were added in wells and incubated with cells for 1 h at 37°C to reaction.

Also, the cell viability was confirmed with an xCELLigence RTDP system (ACEA Biosciences, San Diego, CA, USA). In brief, 2,500 cells/well were grew into E-plates and incubated at 37°C and 5% CO_2_. After 6 hours, media with MWCNTs (0.5 μg/mL) was added into the wells. Then, all cells were incubated for a further 24 h to assess the cell viability. The high-content screening (HCS) multi-120 parameter cytotoxicity analyses were described as the previous study [29]. All tests were measured in triplicate, and the results were averaged.

### Cell cycle and apoptosis analysis

Cell cycle and apoptosis analysis had been performed by flow cytometry (FCM). All cells were harvested with 0.25% trypsin/EDTA (Sigma-Aldrich, Saint Louis, MO, USA) after 24 h treatment and washed with cold PBS. To detect the cell apoptosis, cells were stained with propidium iodide (PI, Sigma-Aldrich, Saint Louis, MO, USA) and annexin V (Sigma-Aldrich, Saint Louis, MO, USA) for 15 min kept in dark place and were analysed by FACS Calibur Flow Cytometry (BD Biosciences, NJ, USA). To detect the cell cycles, cells were fixed with cold 75% ethanol (Sigma-Aldrich, Saint Louis, MO, USA) and stained with PI before quantified by FACS Calibur Flow Cytometry (BD Biosciences, NJ, USA). All tests were measured in triplicate.

### Reactive oxygen species (ROS) detection

Reactive Oxygen Species Assay kit (Beyotime, Haimen, China) was used to detect cellular ROS. The cells were cultured in 10 cm dishes and treated with 0 and 0.5 μg/mL of MWCNTs. After 24 h treatment, cells were harvested and co-incubated with 2′,7′-dichlorofluorescin diacetate (DCFH-DA) for 20 min, and washed thrice with PBS and detected using FCM. Each experiment was performed at least twice.

### Western blot assay

Total protein of cell line lysate (including control and 0.5 μg/mL of MWCNTs groups) was collected, and then separated on SDS-PAGE and transferred to polyvinylidene fluoride membranes (Millipore, Billerica, MA, USA). The antibodies used were anti-CDK4 (Cell Signaling Technology, 1:1000, Danvers, MA, USA), anti-Cyclin D1 (Cell Signaling Technology, 1:1000, Danvers, MA, USA), anti-Cyclin D3 (Cell Signaling Technology, 1:1000, Danvers, MA, USA), anti-P_53_ (Cell Signaling Technology, 1:1000, Danvers, MA, USA), anti-β-actin (Beyotime, 1:1000, China). The immune complexes were detected by enhanced chemiluminescence (Millipore, Billerica, MA, USA). Blots were quantified by densitometry and normalized by use of β-actin to correct for differences in loading of the proteins. For densitometric analyses, the band was quantified using Image Lab software (BioRad laboratories, Hercules, CA, USA). Each experiment was performed at least twice.

### The uptake and distribution of MWCNTs in spermatocyte cell line

After incubating in 6 well plates and treating with 0.5 μg/mL MWCNTs for 24 h, cells were harvested with 0.25% trypsin/EDTA and fixed in 2.5% glutaraldehyde. Then cells were stained with lead citrate and uranyl acetate. Transmission electron microscopy (TEM) was used to detect the uptake and distribution of MWCNTs in cells.

### Long extension-polymerase chain reaction

Long extension polymerase chain reaction (LX-PCR) was performed as previously described [18]. In brief, genomic DNA in cells from each group was isolated with the DNA Isolation Kit (QIAGEN, 56304, Dusseldorf, German). The quantitation of the purified genomic DNA, as well as that of PCR products, was performed fluorometrically using the Quant-iT™ DNA Assay Kit (Life Technologies, Q-33120, CA, USA). PCR amplified products were performed by measuring fluorescence at 485/530 nm with the microplate reader (TECAN Infinite200 PRO). The four pairs of PCR primers employed in this study are given in Supplemental Table 1. The total input of genomic DNA was 15 ng for each PCR reaction. To amplify the long fragment of nDNA (8.7 kb), the thermocycler profile included initial denaturation at 94°C for 1 min, 25 cycles of 94°C 15 s, and 65°C for 12 min, with final extension at 72°C for 10 min. For amplification of a long fragment of mtDNA (10.0 kb), the standard thermocycler program included initial denaturation at 94°C for 1 min, 20 cycles of 94°C for 15 s, and 65°C for 12 min, with final extension at 72°C for 10 min. DNA damage was quantified by comparing the relative efficiency of amplification of large fragments of DNA (8.7 kb from nDNA and 10.0 kb from mtDNA) and normalizing this to the amplification of smaller (6.5 kb and 117 bp) fragments. Total DNA concentration was determined with Quant-iT™ DNA Assay Kit. Meanwhile, partial products were separated on a 2% agarose gel, stained with ethidium bromide. Each sample was run in triplicate, and each experiment was performed at least twice.

### ATP content assay

ATP concentrations were determined using a luciferase-luciferin ATP Assay Kit (Beyotime, Haimen, China) according to the manufacturer's instructions. Protein concentration of the cells was measured using the BCA protein assay, and ATP content was adjusted to the protein concentration, resulted as nmol/mg protein. Each experiment was performed at least thrice.

### Quantitative RT-PCR

Total RNA was extracted from cells including control and MWCNTs treatment group using Trizol (Invitrogen, Carlsbad, CA), according to the manufacturer's instructions. Reverse transcription was performed using a Prime Script RT reagent kit (Takara, Dalian, China).

Real-time PCR reactions were performed with gene-specific primers and SYBR Green PCR Master Mix reagent kits (Takara, Dalian, China) using the ABI 7900 HT fast real-time system (Applied Bio systems, Foster City, CA). The specific primers were listed in Supplemental Table 1. All real-time PCR reactions were performed in triplicate, and the relative quantification in gene expression was determined using the ΔΔCt method, with calibration to control samples [30]. The housekeeping gene *18S rRNA* was used as an internal control. The levels of the genes were normalized relative to the expression levels of the gene *18S rRNA*. Each experiment was performed at least thrice.

### Oxygen consumption rate (OCR) measurements

Cellular oxygen consumption was measured using a Seahorse XF96 Extracellular Flux analyzer (Seahorse Bioscience, North Billerica, MA). In briefly, cells were seeded in Seahorse XF96 cell culture microplates at a density of 1 × 10^4^ cells per well and treated with 0, and 0.5 μg/mL for 24 h. Then, mitochondrial complex inhibitors were injected to all treatments sequentially in the following order: oligomycin (1 μM), carbonyl cyanide-ptrifluoromethoxyphenylhydrazone (FCCP; 0.75 μM), and antimycin A/rotenone (0.5 μM each), and the readings were taken after each inhibitor. OCR was automatically recorded by XF96 software. Rates were calculated per protein levels for control and MWCNTs treatment group.

### Data analysis

Values are expressed as mean ± standard deviation of the mean (S.D.) for all experiments. Non-parametric test and Kruskal-Waillis multiple comparison tests were used to analysis statistically significant differences between the treatments and the control. Statistical Analysis Systems statistical software package version 9.2 (SAS Institute, Inc.) was performed for all statistical analyses. A *P* value < 0.05 was designated as the cut-off for statistical significance. Bonferroni correction and FDR (false discovery rate) correction were performed to counteract the problem of multiple comparisons.

## SUPPLEMENTARY MATERIAL TABLE AND FIGURE


