# Supplementary material for: Toxicological assessment of multi-walled carbon nanotubes in vitro: potential mitochondria effects on male reproductive cells
Source: Oncotarget. 2016 May 29;7(26):39270–8. doi: 10.18632/oncotarget.9689 (PMC5129931; doi:10.18632/oncotarget.9689)
Supplement: Supplementary file 1 [file oncotarget-07-39270-s001.pdf]

Toxicological assessment of multi-walled carbon nanotubes *in vitro*: potential mitochondria effects on male reproductive cells

Supplementary Material

Supplement table 1: Primers used in gene expression analysis

Primers	Sequence
mt-Nd1 F	5’-TCCGAGCATCTTATCCACGC-3’
mt-Nd1 R	5’-GTATGGTGGTACTCCCGCTG-3’
mt-Nd2 F	5’-ATCCTCCTGGCCATCGTACT-3’
mt-Nd2 R	5’-ATCAGAAGTGGAATGGGGCG-3’
mt-Nd3 F	5’-TTGCATTCTGACTCCCCCAA-3’
mt-Nd3 R	5’-GACGTGCAGAGCTTGTAGGG-3’
mt-Nd4 F	5’-TAATCGCACATGGCCTCACA-3’
mt-Nd4 R	5’-GCTGTGGATCCGTTCGTAGT-3’
mt-Nd4l F	5’-TAGTATATCGCTCACACCTC-3’
mt-Nd4l R	5’-GTAGTCTAGGCCATATGTG-3’
mt-Nd5 F	5’-TAACCGCATCGGAGACATCG-3’
mt-Nd5 R	5’-GTGGAGGCCAAATTGTGCTG-3’
mt-Nd6 F	5’-GGATTGGGGTAGCGGCAATA-3’
mt-Nd6 R	5’-CCGCAAACAAAGATCACCCA-3’
mt-Cytb F	5’-ACGCAAACGGAGCCTCAATA-3’
mt-Cytb R	5’-CCTCATGGAAGGACGTAGCC-3’
mt-Co1 F	5’-GCTAGCCGCAGGCATTACTA-3’
mt-Co1 R	5’-CTCCTCCAGCGGGATCAAAG-3’
mt-Co2 F	5’-AACCGAGTCGTTCTGCCAAT-3’
mt-Co2 R	5’-CTAGGGAGGGGACTGCTCAT-3’
mt-Co3 F	5’-AGCCTTTTCAGCCCTCCTTC-3’
mt-Co3 R	5’-GTGGCCTTGGTAGGTTCCTT-3’
mt-Atp6 F	5’-GCAGTCCGGCTTACAGCTAA-3’
mt-Atp6 R	5’-GGTAGCTGTTGGTGGGCTAA-3’
mt-Atp8 F	5’-ACAAACATTCCCACTGGCAC-3’
mt-Atp8 R	5’-TTGGGGTAATGAATGAGGCAA-3’
18S ribosomal RNA F	5’- GGGAGCCTGAGAAACGGC-3’
18S ribosomal RNA R	5’-GGGTCGGGAGTGGGTAATTT-3’
8.7-kb fragment F	5′-TTGAGACTGTGATTGGCAATGCCT-3′
8.7-kb fragment R	5′-CCTTTAATGCCCATCCCGGACT-3′
6.5-kb fragment F	5′-TATCTCTCTTCCTCTTCACTTCTCCCCTGG-3′
6.5-kb fragment R	5′-CGTGATGCCGCCGTTGAGGGTCTCCTG-3′
10-kb mitochondria fragment F	5′-GCCAGCCTGACCCATAGCCATATTAT-3′
10-kb mitochondria fragment R	5′-GAG AGATTTTATGGGTGTATTGCGG-3′
117-bp mitochondria fragment F	5′-CCC AGCTACTACCATCATTCAAGT-3′

117-bp mitochondria fragment	R	5′-GATGGTTTGGGAGATTGGTTGATG-3′

F: forward; R: reverse

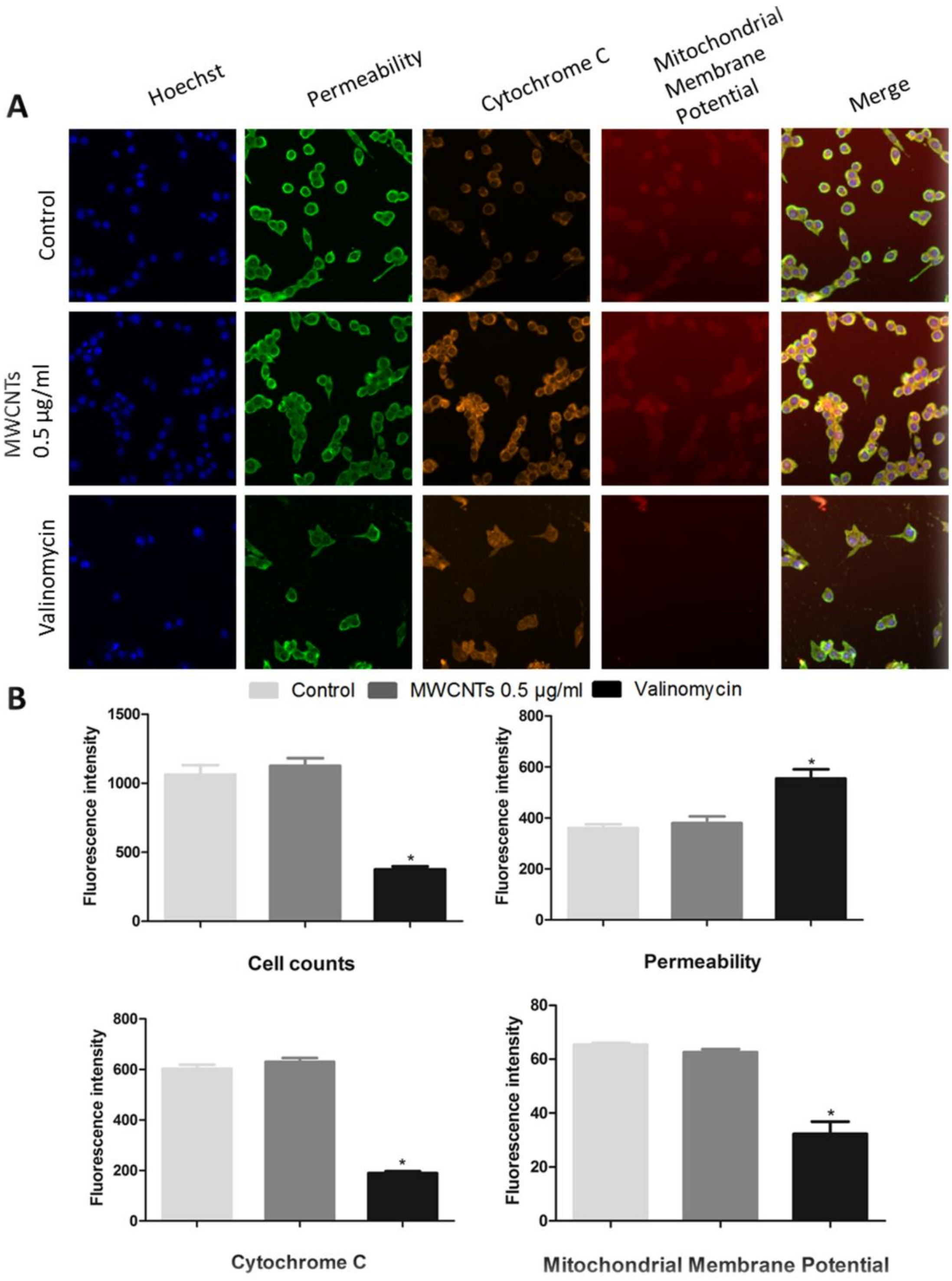

Figure S1: High content screening (HCS) assays. The images (A) and values (B) of permeability, mitochondrial membrane potential and cytochrome c in GC-2spd cells were captured and analyzed by HCS after MWCNTs exposure. Significance indicated by: *$p < 0.05$, versus control.
